# Anti-malarial and haematological evaluation of the ethanolic, ethyl acetate and aqueous fractions of *Chromolaena odorata*

**DOI:** 10.1186/s12906-023-04200-8

**Published:** 2023-11-09

**Authors:** Tobiloba Christiana Elebiyo, Olarewaju Michael Oluba, Oluyomi Stephen Adeyemi

**Affiliations:** 1 SDG 03 Group – Good Health & Well-being, Landmark University, Omu-Aran, 251101 Kwara State Nigeria; 2 Department of Biochemistry, Landmark University, PMB 1001, Omu-Aran, 251101 Nigeria; 3 Present Address: Department of Biochemistry, Laboratory of Medicinal Biochemistry, Nanomedicine, & Toxicology, Bowen University, Iwo, Nigeria; 4 Laboratory of Sustainable Animal Environment, Graduate School of Agricultural Science, Tohoku University, 232-3 Yomogida, Naruko-Onsen, Osaki, Miyagi 989-6711 Japan

**Keywords:** Botanical drug, Haematology, Malaria treatment, Medicinal biochemistry, *Plasmodium berghei*

## Abstract

Malaria is a global health challenge with endemicity in sub-Saharan Africa, where there are multiple drug-resistant strains and limited access to modern health care facilities, especially in rural areas. Studies indicate that African traditional medicine could make a substantial contribution to the reduction of malaria-related deaths and achievement of universal health coverage (UHC), particularly in these regions. Thus, this study evaluated the curative antimalarial effects of *Chromolaena odorata* leaf extract using mouse model. Forty-five (45) albino mice weighing between 18 and 22 g were grouped into nine groups of 5 animals each. Animals in groups 2–9 were infected with the chloroquine-resistant strain of *Plasmodium berghei,* while animals in groups 3–9 were subsequently treated with 10 mg/kg chloroquine, a combination of 1.4 mg/kg artemether and 8.75 mg/kg lumefantrine (Coartem), and varying concentrations of the fraction from the aqueous leaf extract of *C. odorata* at day 3 post-infection. The findings from this study indicate that treatment with 400 mg/kg of the ethanolic fraction of the crude extract resulted in a significant decrease in parasite load (97.6%), which was comparable to the activities of the conventional drugs chloroquine (98.6%) and Coartem (98.8%). The ethyl acetate and ethanolic fractions at 400 mg/kg also ameliorated the significant alterations in the red blood cells, white blood cells, and platelets of the infected animals. The high antimalarial activity displayed by the ethanolic fraction could be due to the presence of quercetin and kaempferol, as detected by high performance liquid chromatography (HPLC) analysis. The findings suggest that the fractions from *C. odorata* could serve as an alternative source of malaria therapy, particularly in sub-Saharan Africa.

## Introduction

Despite considerable advancements in the development of antimalarial therapies, the worldwide burden of malaria persists, as the disease incidence increased from 245 million cases in 2020 to 247 million cases in 2021 [1–3]. Regions of sub-Saharan Africa are most plagued by malaria; this is primarily due to the climatic conditions in this region, which foster the ideal habitat for the parasite's vector, female Anopheles mosquitoes, to reproduce and propagate. In addition, there has been an increase in parasite resistance to antimalarial drugs and vector resistance to insecticides and treated nets, thereby leading to high mortality, particularly among pregnant women and young children [1]. Four sub-Saharan nations—Nigeria, the Democratic Republic of the Congo, the United Republic of Tanzania, and Niger—accounted for 51.9% of malaria-related deaths in 2021 [3].

The fact that two effective antimalarial drugs, quinine, and artemisinin, were discovered in plants provides further proof of the significance of plants and natural products in the fight against malaria. According to Soni et al. [4] and Kumar et al. [5], traditional medicine is a significant and sustainable strategy to treat malaria, especially in low-income regions where drug resistance is common and access to effective anti-malarial drugs is limited within this region. Kasilo et al. [6] also highlighted the possibility of African traditional medicine making a substantial contribution to the achievement of universal health coverage (UHC), particularly in rural areas with limited access to modern healthcare facilities.

One of the adverse effects of malarial infection is the alteration in haematological parameters as studies have reported prevalence of malaria-associated anaemia [7–9]. Recent studies on malaria infected patients have linked the disease to alterations in haemoglobin concentration and red blood cell counts, due to the parasite’s ability to haemolyze red blood cells and suppress erythropoiesis in vertebrate hosts [8, 10]. Experimentally, alterations in haematological parameters such as, percentage haematocrit, mean corpuscular volume, red blood cell and white blood counts have established in malaria-infected mice models [11–13].

Some of the major plants identified by ethno-medicinal studies as anti-malarials, particularly in Africa, are *Cymbopogon citratus* (S), *Morinda lucida* L*., Azadiratchta indica, Psidium guajava, Citrus aurantifolia, Vernonia amygdalina, Magnifera indica* L*., Persea americana* Mill*., Allium sativum, Jatropha curas, Allium cepa, and Cassia fistulosa* [14–18]. A few ethnobotanical studies, particularly from Nigeria, also identified the unwanted weed *Chromolaena odorata (L.) R.M. King & H. Rob.,* as a plant with potential antimalarial properties [16, 17].

A plethora of studies has validated the anti-malarial activities of different plants in mice models [19–23]. According to Nworgu et al. [23], the ethanolic extract of combined *Cassia sieberiana and Chromolaena odorata* significantly reduced parasitemia in mice infected with the chloroquine sensitive strains of *Plasmodium berghei.* Plant extracts from *Cuscuta reflexa, Maesa lanceolata* and *Carica papaya* [19–21]. In addition, treatment of malaria with extracts from plants such as *Salacia nitida*, *Artemisia annua,* and *Senna alata*, have been reported to reverse the haematological alterations associated with malaria infection in mice models [13, 24, 25]. However, fewer studies have validated the anti-malarial properties of *C. odorata* [22, 23]. Therefore, the present study evaluated the curative antimalarial properties of *C. odorata* in a mouse model of malaria. In addition, we identified the chemical constituents of the most potent antimalarial fraction using HPLC–UV.

## Materials and methods

### Plant preparation and extraction

**Fig. 1:**
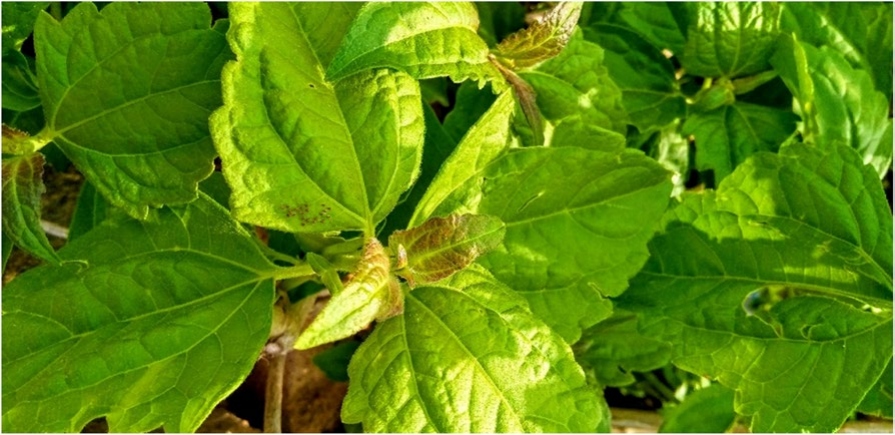
*Chromolaena odorata* (L.) R.M. King & H. Rob

Fresh *C. odorata* leaves (Fig. 1) were collected from a botanical garden in Ilorin, Kwara State, between March and June 2021. The leaves were authenticated by Mr. Bolu Ajayi, at the Department of Plant Biology, Faculty of Life Sciences, University of Ilorin, Nigeria. The authentication was registered under voucher number UILH/001/1281/2021. The name of the species was fully validated on http://mpns.kew.org/mpns-portal/?_ga=1.111763972.1427522246.1459077346. The leaves of *C. odorata* were harvested, washed with distilled water, and air-dried for 2 weeks. The evenly dried leaves were milled to a fine powder using an electrical grinder (Excella mixer grinder by Kanchan International Limited, Dabhel, Daman-396210).

#### Crude extraction

To simulate the traditional use of the leaves of *C. odorata* in the treatment of malaria in Nigeria, a crude extract of the leaves was obtained via aqueous extraction following the methods described by Yakubu et al. [26] and Sadiq et al. [27]. Using a solid to solvent ratio of 1:10 (w/v), 1000 g of the powdered *C. odorata* leaves were macerated in 10 L of distilled water and agitated for 24 h on a mechanical shaker at 200 rpm at 25 °C. The mixture was filtered using Whatman filter paper (No. 1), and the resulting filtrate was lyophilized in a freeze dryer (Mini Lyrotrap, LTE Scientific Limited, Greenfield, Oldham, United Kingdom).

#### Solvent partitioning

Solvent portioning was achieved following the method described by Zeleke et al. [28], with slight modifications. In brief, 180 g of crude aqueous extract was reconstituted in 450 mL of distilled water and stirred until a homogenous solution was obtained. The reconstituted aqueous extract was then transferred into a 1000 mL separating funnel, and 500 mL of 96.5% n-hexane was subsequently added and left to stand for 12 h to de-fat the crude extract. After 12 h, the aqueous layer was eluted, and the fatty layer was decanted and discarded. This process was repeated once, after which the defatted crude aqueous extract was transferred into a clean separating funnel, and solvent–solvent partitioning was carried out by using two solvents with different polarities: 100% ethyl acetate (4.4 polarity) and 96.5% ethanol (6.1 polarity).

Based on the polarities, the ethyl acetate fraction was obtained first, the defatted crude extract was transferred into a 1000 mL separating funnel, and 600 mL 100% ethyl acetate was added. The mixture was agitated and left for 12 h, after which the aqueous residual fraction (lower layer) and the ethyl acetate fraction (upper layer) were eluted into different beakers. This process was repeated by adding 400 mL of ethyl acetate to the aqueous residual fraction. After obtaining the ethyl acetate fraction, the ethanolic fraction was obtained using the same procedure. The three different fractions, the aqueous residual fraction (ARF), ethyl acetate fraction (EAF), and ethanolic fraction (EF), were concentrated at 40 °C using a water bath, weighed, and stored at 4 °C until further use.

### Experimental animals

Forty-five (45) mice and five (5). Wistar albino rats were obtained from the animal holding facility of the Institute for Advanced Medical Research and Training (IAMRAT), University of Ibadan, Ibadan, Oyo state, Nigeria, after receiving ethical approval (LUAC/BCH/2022/005A) from the Landmark University Omu-Aran, Kwara State, Nigeria. The animals received humane care in compliance with the institution’s guidelines and criteria outlined in the National Institute of Health (NIH) Guidelines for the care and use of laboratory animals [29]. The animals were kept in well-ventilated plastic cages and housed at 27 °C and a 12-h day/night cycle. The experimental animals were fed commercial rat pellets and were given unlimited access to clean water.

### Inoculum

Chloroquine-sensitive strains of *P. berghei* (ANKA) were obtained from the Institute for Advanced Medical Research and Training (IAMRAT), University College Hospital, Ibadan, Oyo state, Nigeria. Parasitized erythrocytes were obtained from an anaesthetized donor-infected mouse via cardiac puncture into a plain bottle containing acid citrate dextrose (ACD) and were serially diluted with normal saline to make a suspension containing 1 × 10^7^ parasitized erythrocytes in every 2 mL of infected blood.

### Standard drugs

The standard antimalarial drugs chloroquine phosphate (250 mg) and Coartem (20/120 mg Artemether and Lumefantrine) were manufactured by Jopan Pharmaceuticals Limited, Nigeria and Tuyil Pharmaceutical Limited, Nigeria, respectively.

### In vitro quantitative phytochemical screening of the crude extract and solvent fractions of *C. odorata*

In vitro quantitative phytochemical screening of *C. odorata* aqueous extract, aqueous residual fraction, ethanolic and ethyl acetate fractions were evaluated by determining the total flavonoid and phenolic content; total flavonoid content; total antioxidant capacity; ferrous reducing capacity; nitric oxide scavenging capacity; and 2,2-diphenyl-1-picrylhydrazy (DPPH) radical scavenging power using the methods described by Elebiyo et al. [30]. To evaluate the In vitro bioactivity of the crude extract and fractions, stock solutions of the crude extract and fractions were prepared by dissolving 6 mg of the dried sample in 1 mL of dimethyl sulfoxide (DMSO). Varying concentrations of the plant extract (60–900 µg/mL) were then prepared from the stock solutions.

### Lethality study and dose determination

The lethal dose of the crude aqueous extract of *C. odorata* was first established using five female Wistar rats following the up and down procedure described in OECD guideline 425 [31]. The selection of doses for the treatment was later calculated as one-fifth and one-twentieth of the LD_50_ (2000 mg/kg) using the formula presented below:

$$High\,dose=\frac15\ast2000\;{mgkg}^{-1}=400\;{mgkg}^{-1}$$

$$Low\,dose=\frac1{20}\ast2000\;{mgkg}^{-1}=100\;{mgkg}^{-1}$$

### In vivo curative antimalarial screening of solvent fractions of the aqueous leaf extract of *C. odorata*

The antimalarial potential of the solvent fractions from the aqueous leaf extract of *C. odorata* was screened using a curative malarial test described by Baah et al. [32]. This was done to ascertain that the *C. odorata* leaves possess anti-malarial properties.

#### Experimental design and grouping

Forty-five (45) albino mice weighing between 18 and 22 g were used for curative antimalarial screening of the ethyl acetate (EAF), ethanolic (EF) and aqueous residual (ARF) fractions of the aqueous leaf extract of *C. odorata* using the treatment grouping presented in Table 1. Forty of the experimental animals were inoculated intraperitoneally with 0.2 mL of infected blood suspension. Seventy-two hours after inoculation with the parasite, parasitemia of the infected mice was established using blood smear microscopy. The infected animals were randomly distributed into eight (8) groups (Groups 2–9). The animals in group 2 served as the negative control group and received no form of treatment with the fractions or standard drugs throughout the experiment. From day 3 post-infection, the animals in groups 3–9 received oral administration of the different doses of fractions or standard drugs for 4 days. The animals in groups 3 and 4 served as the positive control groups and were treated with 10 mg/kg chloroquine and a combination of 1.4 mg/kg artemether and 8.75 mg/kg lumefantrine (Coartem), respectively. Group 5 animals received 100 mg/kg of the aqueous residual fraction, while groups 6 and 7 received 100 mg/kg and 400 mg/kg of the ethyl acetate fraction, respectively. Groups 8 and 9 received 100 mg/kg and 400 mg/kg ethanolic fraction, respectively (Table 1).

**Table 1:** Experimental grouping

**S/n**	**Code**	T**reatments (*n* = 5 mice per group)**
**1**	Group 1	Normal control (No infection + 2.5% DMSO)
**2**	Group 2	Negative control* (Plasmodial* Infection + 2.5% DMSO)
**3**	Group 3	Standard control 1 (*Plasmodial* Infection + 10 mg/kg of Chloroquine in 2.5% DMSO)
**4**	Group 4	(Standard control 2 (*Plasmodial* Infection + 1.4 mg/kg artemether + 8.75 mg/kg Lumefantrine in 2.5% DMSO)
**5**	Group 5	*Plasmodial* Infection + 100 mg/kg of ARF in 2.5% DMSO
**6**	Group 6	*Plasmodial* Infection + 100 mg/kg of EAF in 2.5% DMSO
**7**	Group7	*Plasmodial* Infection + 400 mg/kg of EAF in 2.5% DMSO
**8**	Group 8	*Plasmodial* Infection + 100 mg/kg of EF in 2.5% DMSO
**9**	Group 9	*Plasmodial* Infection + 400 mg/kg of EF in 2.5% DMSO

*ARF* aqueous residual fraction, *EAF* ethyl acetate fraction, *EF* ethanolic fraction

#### Microscopic determination of parasite burden in the blood

Parasitaemia levels in the mice were determined using a slightly modified method described Ounjaijean et al. [33]. Blood from the tails of the experimental mice at days 0, 3, 5, and 7 post-malarial infection was smeared on clean glass slides to make a thin film. The slides were then fixed with a few drops of methanol, air-dried and stained with 10% Giemsa solution prepared with phosphate buffer (pH 7.2). The slides were then air-dried for 30 min and rinsed with distilled water to remove excess stain and then air-dried for another 20 min before microscopic examination. The parasite burden in the blood of each mouse was evaluated from 5 randomly selected viewing fields under a magnification of × 100 objective lens using a light microscope. The percentage parasite burden in the blood and percentage mean parasitemia suppression were determined using the formula described by Baah et al. [32]:

$$\%\;parasite\;burden=\frac{number\;of\;parasitized\;RBC}{total\;number\;of\;RBC\;counted}\ast100$$

$$\%\;mean\;parasitemia\;suppression=\frac{\%\;parasitemia\;in\;negative\;control-\%\;mean\;parasitemia\;in\;treatment\;groups}{\%\;mean\;parasitemia\;in\;NC}\ast100$$

### Haematological screening

The full blood count of the infected animals was performed on the 7^th^ day post-infection using an automated haematology analyser. Parameters evaluated included white blood cell (WBC), red blood cell (RBC), haemoglobin (HGB) and haematocrit (HCT), mean corpuscular volume (MCV), mean corpuscular haemoglobin concentration (MCHC), red blood cell distribution width -CV (RDW-CV), red blood cell distribution width-SD (RDW-SD), platelet (PLT), mean platelet volume (MPV), Platelet Distribution Width (PDW), plateletocrit (PCT), lymphocytes (LYMPH) and neutrophils (NEUT).

### High-performance liquid chromatography (HPLC) analysis

The phytochemical constituents of the most potent fraction of the crude extract were quantified by HPLC coupled to ultraviolet spectroscopy using the procedure described by Farag et al. [34]. A 99.8% acetonitrile (Sigma Aldrich. Burlington, Massachusetts, United States), and water were used as the mobile phase for elution on a µBONDAPAK C18 column (3.9 mm × 300 mm, 10-µm particle size, 70:30). The phytochemical constituents of 10.0 g of the ethanolic fraction of *C. odorata* were extracted using acetonitrile and stabilized using 99.7% ethyl acetate (Sigma Aldrich. Burlington, Massachusetts, United States), after which 5 µL was injected into the system and monitored at a flow rate of 2 mL/min. The UV detection was performed at 330 nm while peaks were labelled based on individual compounds' retention time using the Phytochemical Metabolite Library of Standards by IROA Technologies, with the technical support of Bato Chemical Laboratories Limited, Lagos State Nigeria (https://batochemlab.com/).

#### Data analysis

All data were analysed using GraphPad Prism 9.3. Descriptive statistics were used to examine the trends in each sample or experimental groups while one-way and two analysis of variances were used to establish the relationship across the different treatment groups. The variations in the observations across group were considered significant at *p* < 0.05 and 95% confidence level.

## Results

### Quantitative phytochemical screening of the crude extract and fractions of *C. odorata*

#### Total phenolic and total flavonoid contents

**Fig. 2:**
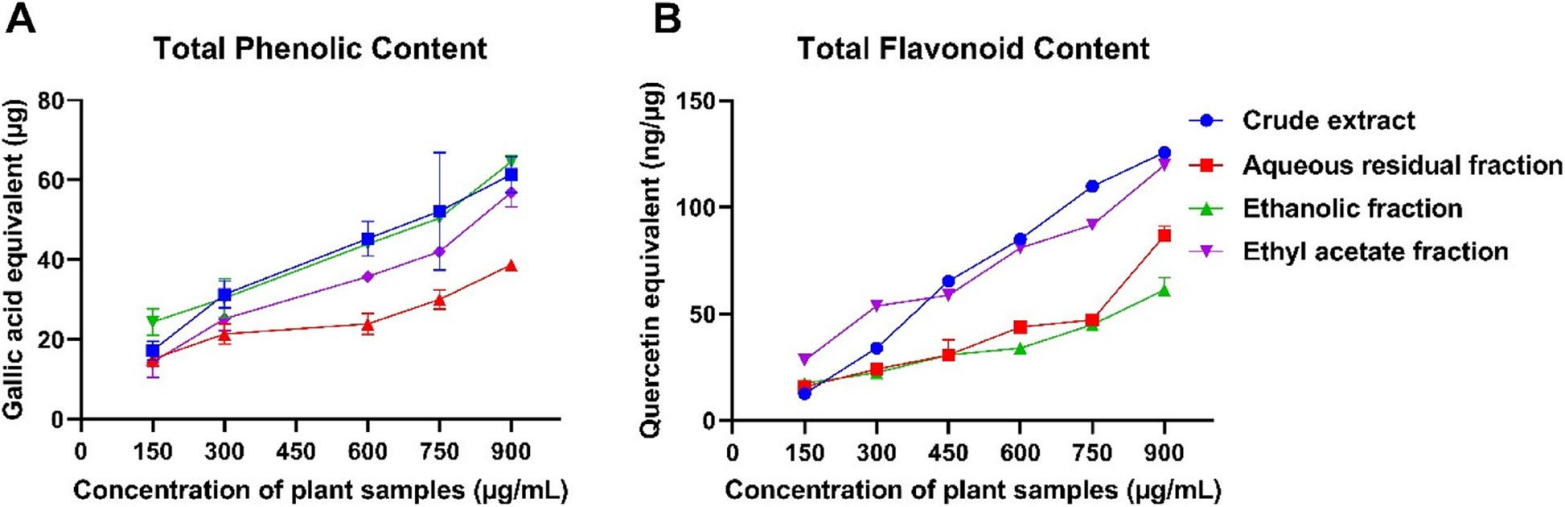
Total phenolic and flavonoid contents of the crude aqueous extract and fractions of *C. odorata*. The data are the mean of three replicates ± standard deviation (SD)

Phytochemical screening of the crude aqueous leaf extract of *C. odorata* and its solvent fractions revealed that the concentrations of flavonoids and phenolic compounds in the plant samples increased in a dose-dependent manner (Fig. 2). The crude extract and the EF and EAF of *C. odorata* had comparative phenolic contents of 6.82 ± 0.5, 7.18 ± 0.08 and 6.3 ± 0.56 g gallic acid equivalent (GAE)/100 g extract, respectively. ARF had the lowest phenolic content of 4.29 ± 0.06 g GAE/100 g extract. The total flavonoid content of the extract was 13.96 ± 0.11 g quercetin equivalent (QUE)/100 g extract in CE, 9.63 ± 0.51 g QUE/100 g extract in ARF, 13.29 ± 0.52 g QUE/100 g in EAF and 6.79 ± 0.64 g QUE/100 g in EF.

#### Total antioxidant capacity and ferric reducing power

**Fig. 3:**
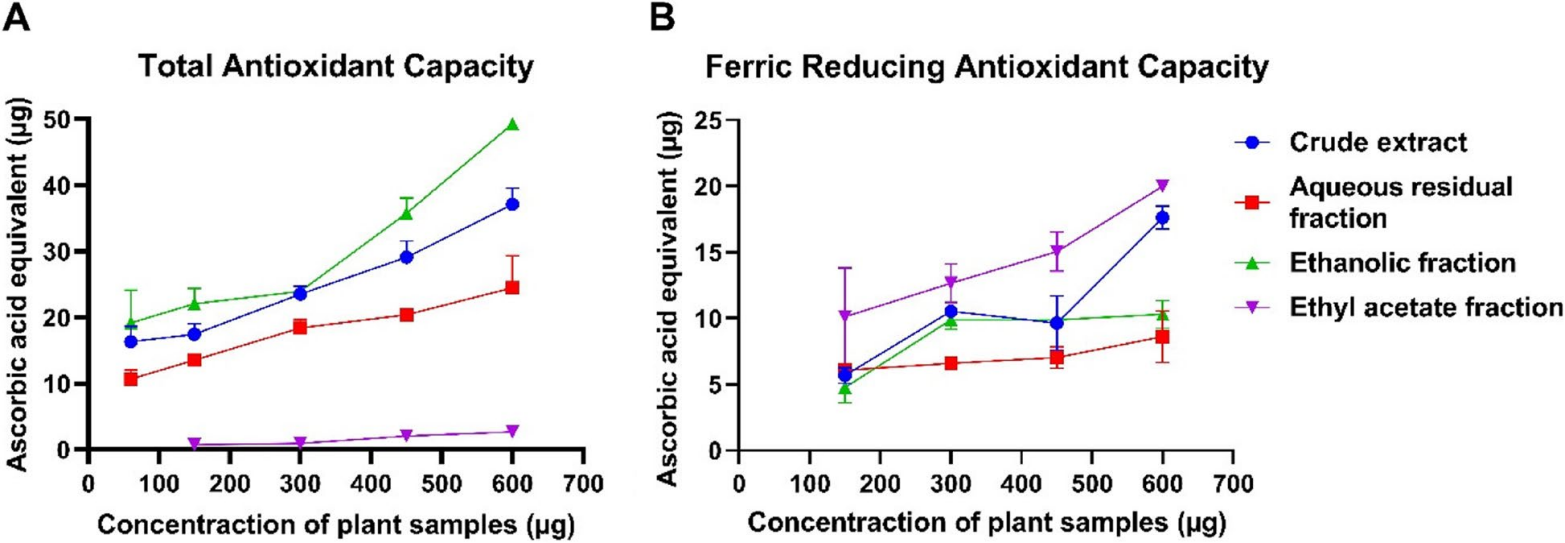
Total antioxidant capacity and ferric antioxidant capacity of the crude aqueous extract and fractions of *C. odorata*. The data are the mean of three replicates ± standard deviation (SD)

The total antioxidant and ferric reducing antioxidant capacities of the crude extract and solvent fractions of *C. odorata* increased in a dose-dependent manner. As shown in Fig. 3, EF showed the highest total antioxidant capacity of 8.21 ± 0.07 g AA/100 g extract, followed by the crude extract, 6.19 ± 0.4 g AA/100 g extract; ARF, 4.08 ± 0.81 g AA/100 g extract; and EAF, 0.45 ± 0.05 g AA/100 g extract. In contrast, the crude extract and EAF showed comparative ferric antioxidant capacities of 2.93 ± 0.14 and 3.32 ± 0.038 g AA/100 g extract, respectively, while those of ARF and EF were lower at 1.43 ± 0.32 and 1.72 ± 0.17 g AA/100 g extract, respectively.

#### Scavenging properties for DPPH and nitric oxide free radicals

**Fig. 4:**
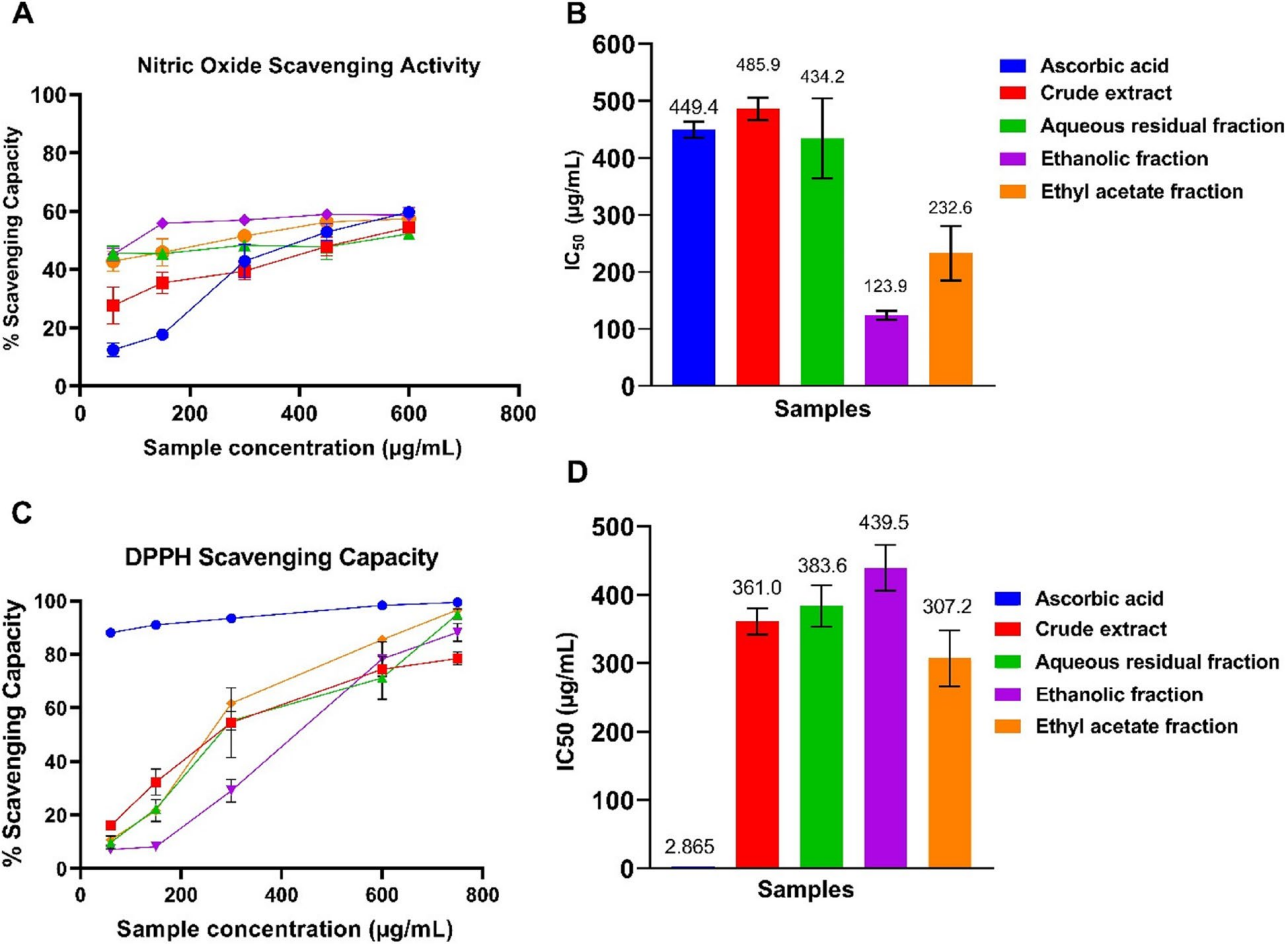
DPPH and nitric oxide scavenging power of the crude aqueous extract and fractions of *C. odorata*. The data are the mean of three replicates ± standard deviation (SD)

The DPPH scavenging power of the crude aqueous extract of *C. odorata* (74.41 ± 2.45% at 750 µg/mL) was significantly lower (*p* = 0.009) than that of ascorbic acid (99.44 ± 0.320% at 750 µg/mL). However, there was no significant difference in the DPPH scavenging power of ARF (95.07 ± 1.92, *p* = 0.999), EF (88.25 ± 3.38, *p* = 0.063) and EAF (96.64 ± 1.27, *p* = 0.126) relative to ascorbic acid at 750 µg/mL (Fig. 4). The nitric oxide scavenging power of EF (58.68 ± 2.63%, *p* = 0.999) and EAF (57.51 ± 0.835%, *p* = 0.069) also compared favourably with ascorbic acid (59.71 ± 0.41%) at 600 µg/mL. On the other hand, the NO scavenging properties of the crude extract (54.41 ± 0.95%, *p* = 0.011) and ARF (52.27 ± 1.09%, *p* = 0.008) were significantly lower than that of ascorbic acid.

### Lethality dose of *C. odorata* aqueous leaf extract

The results from the lethality test revealed that 2000 mg/kg of the crude extract did not induce adverse reactions or significant death (80% survival rates) in the experimental animals throughout the 14-day observation period (Table 2).

**Table 2:** Behavioural changes following exposure to 2000 mg/kg bw C. odorata aqueous extract

**Behavioura**l** indices**	**Crude extract (2000 mg/kg bw)**
**Fur, skin**,** and eye discolouration**	None
**Drooling**	None
**Respiratory distress**	None
**Mobility**	Normal
**Sleep**	Normal
**Convulsions and tremors**	None
**Diarrhoe**a	None

### Curative antimalarial activity of the aqueous residual, ethyl acetate and ethanolic fractions of *C. odorata* aqueous leaf extract

**Fig. 5:**
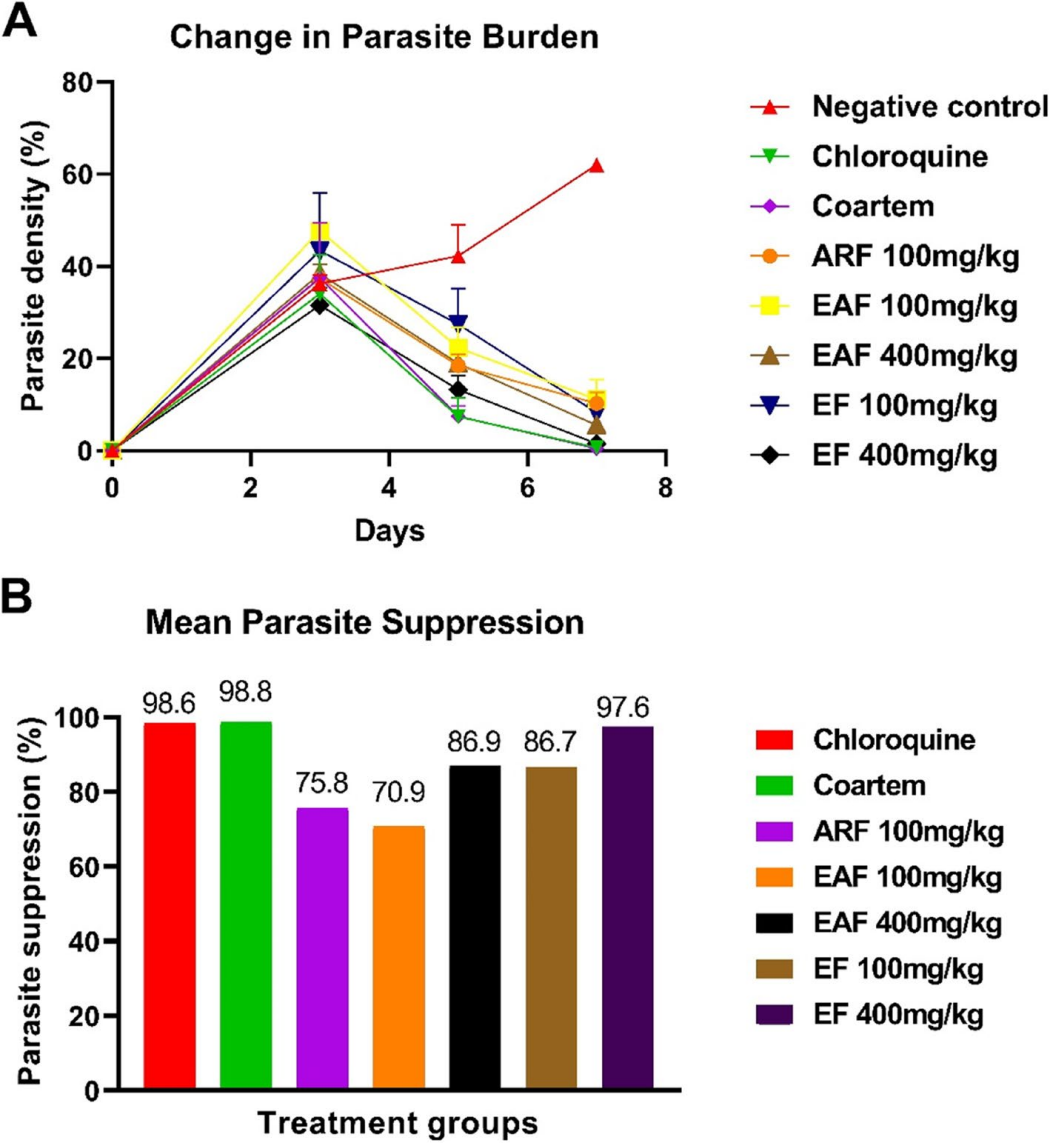
Effect of standard drugs and *C. odorata* fractions on the parasitemia burden of *P. berghei*-infected mice. The data are the mean of three replicates ± standard deviation (SD). ARF represents the aqueous residual fraction; EAF represents the ethyl acetate fraction; EF represents the ethanolic fraction

The data presented in Fig. 5A revealed no significant difference in the parasite burden of all the animals infected with *P. berghei* at day 3 post-infection. However, after a 4-day treatment with the different fractions of *C. odorata* or standard drugs, a significant decrease in parasite burden was observed in the RBCs of infected mice treated with chloroquine (0.6 ± 0.10%, *p* = 0.019), Coartem (0.47 ± 0.29%, *p* = 0.011)), ARF 100 mg/kg (10.23 ± 1.38%, *p* = 0.0007), EAF 100 mg/kg (11.07 ± 2.47%, *p* = 0.0044), EAF 400 mg/kg (5.5 ± 0.50, *p* = 0.0025), EF 100 mg/kg (8.25 ± 1.95%, *p* = 0.024) and EF 400 mg/kg (1.50 ± 0.5%, *p* = 0.003) compared with the negative control (62.0 ± 1.0%). Chloroquine, Coartem and EF 400 mg/kg showed the highest parasite suppression activity of 98.6%, 98.8% and 97.6%, respectively (Fig. 5B). The percentages of mean parasite suppression for the other treatment groups were 75.8%, 70.9%, 86.9% and 86.7% for ARF 100 mg/kg, EAF 100 mg/kg, EAF 400 mg/kg and EF 100 mg/kg, respectively.

### Effect of the aqueous residual, ethyl acetate and ethanolic fraction on the haematological indices of *P. berghei*-infected mice

#### Effect of *C. odorata* fractions on red blood cell indices of malaria-infected mice

**Fig. 6:**
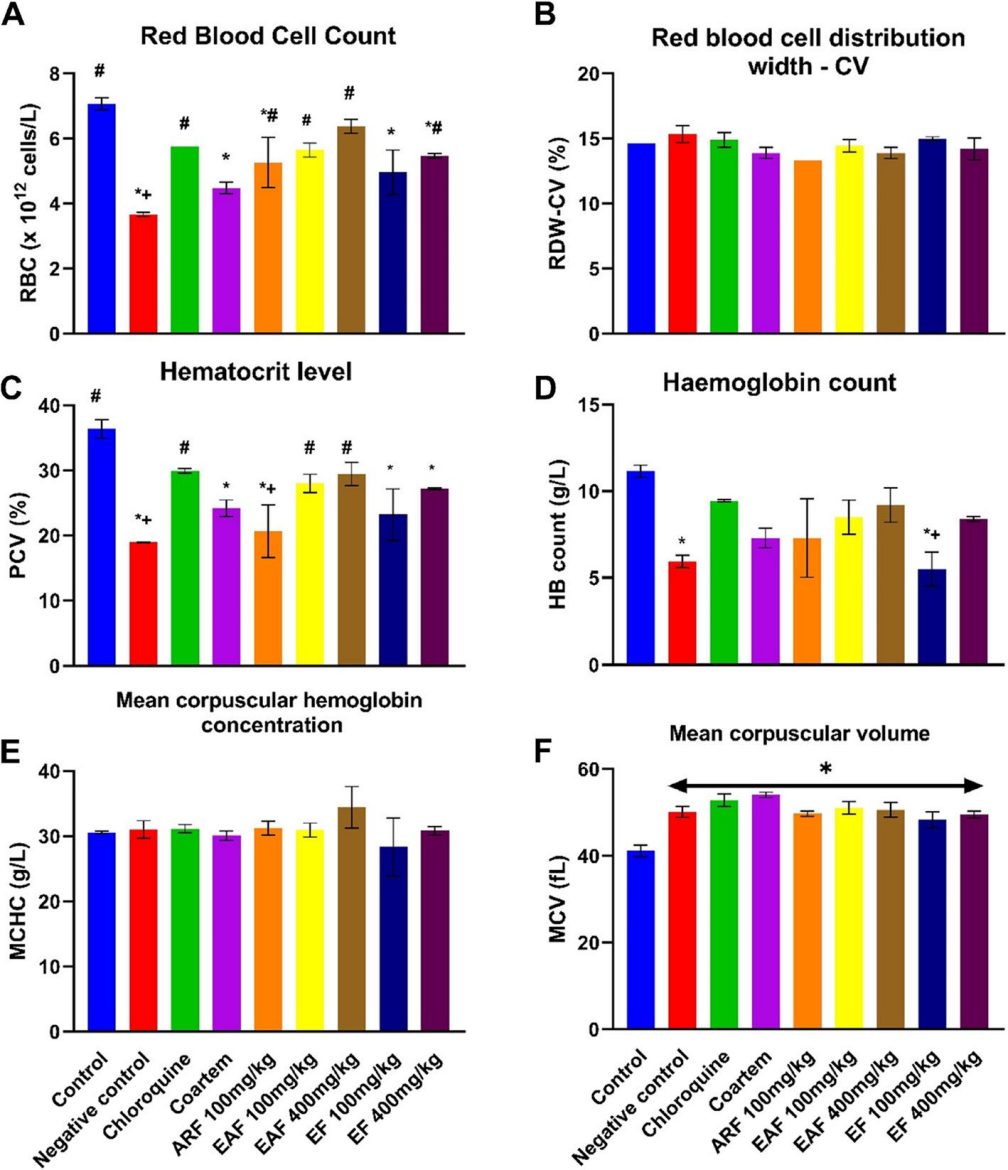
Effect of standard drugs and *C. odorata* fractions on the red blood cell indices of *P. berghei*-infected mice*.* The data are the mean of three replicates ± standard deviation (SD). All measures were considered significant at *p* < 0.05. ^*^ represents outcomes significantly different from control; ^#^represents outcomes significantly different from negative control; ^+^represents outcomes significantly different from chloroquine-treated group; ^&^represents significantly different from Coartem-treated group. ARF represents the aqueous residual fraction; EAF represents the ethyl acetate fraction; EF represents the ethanolic fraction

The data presented in Fig. 6A show that treatment with the *C. odorata* fractions or standard drugs significantly mitigated the effect of malaria infection on the red blood cells of infected mice; the RBC count increased in infected animals treated with chloroquine (5.75 ± 0.00, *p* = 0.0053), ARF 100 mg/kg (5.27 ± 0.77, *p* = 0.03), EAF 100 mg/kg (5.65 ± 0.21, *p* = 0.0079), EAF 400 mg/kg (6.38 ± 0.15, *p* = 0.0008) and EF 400 mg/kg (5.47 ± 0.07, *p* = 0.015) compared with the untreated infected animals (3.67 ± 0.06). There was no significant difference in the RBC count of the noninfected mice (7.070 ± 0.18) and the animals treated with chloroquine (*p* = 0.084), EAF 100 mg/kg (0.058) and EAF 400 mg/kg (*p* = 0.647). In addition, the effect of all the plant fractions on the RBC count of malaria-infected mice also compared favourably with chloroquine.

There was a significant reduction in the haematocrit levels in the untreated animals infected with *P. berghei* (18.95 ± , 0.07, *p* = 0.0004) compared with the uninfected animals (36.40 ± 1.41), as shown in Fig. 6C. This alteration was significantly mitigated by the administration of chloroquine (29.95 ± 0.34, *p* = 0.01), 100 mg/kg EAF (28.00 ± 1.41, *p* = 0.033) and 400 mg/kg EAF (29.45 ± 1.768, *p* = 0.0137). The negative control group (5.95 ± 0.35, *p* = 0.009) also showed a significant decrease in haemoglobin count when compared with the uninfected control group (11.15 ± 0.35). However, there was no significant difference in the haemoglobin count of the animals treated with chloroquine (9.45 ± 0.07, *p* = 0.718), Coartem (7.3 ± 0.57, *p* = 0.052), ARF 100 mg/kg (7.3 ± 2.3, *p* = 0.052), EAF 100 mg/kg (8.5 ± 0.98, *p* = 0.265), EAF 400 mg/kg (9.2 ± 0.99, *p* = 0.582) and EF 400 mg/kg (8.4 ± 0.14, *p* = 0.233) when compared with the uninfected control group (Fig. 6D). The data presented in Fig. 6F indicated that malaria infection caused a significant increase in the mean corpuscular volume in the negative control group (50.1 ± 1.27 *p* = 0.0014) compared with the uninfected control group (41.2 ± 1.34). However, treatment with standard drugs or the fractions of the aqueous extract of *C. odorata* did not lead to any significant ameliorative effect on the MCV of the infected animals. In addition, there was no significant alteration in the red blood cell distribution width –CV (RDW-CV) and mean corpuscular haemoglobin concentration (MCHC) of all the animals in the treatment groups when compared with the control (Fig. 6B and E).

#### Effect of *C. odorata* fractions on white blood cell indices of malaria-infected mice

**Fig. 7:**
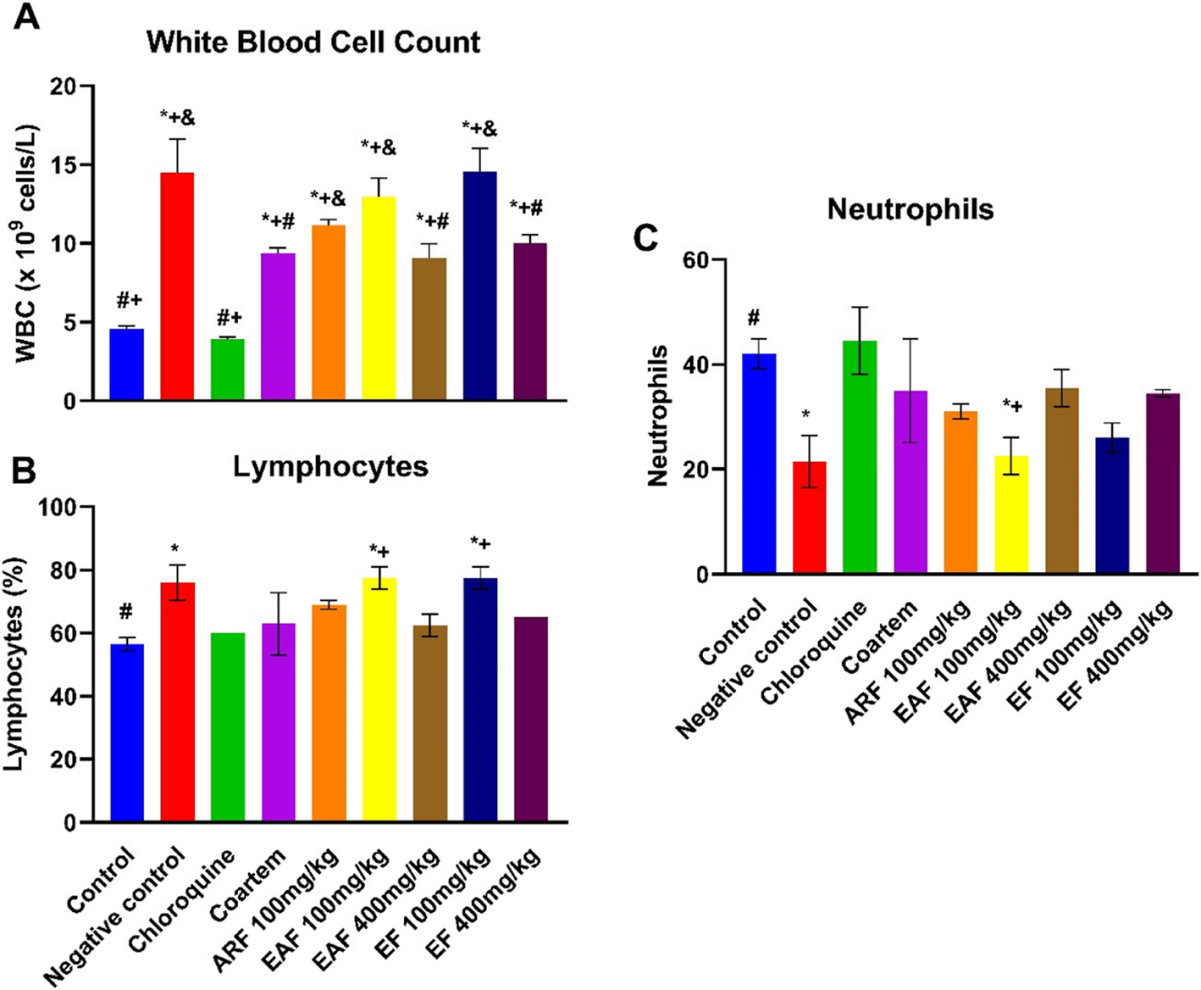
Effect of standard drugs and *C. odorata* fractions on the white blood cell indices of *P. berghei*-infected mice*.* The data are the mean of three replicates ± standard deviation (SD). All measures were considered significant at *p* < 0.05. ^*^ represents outcomes significantly different from control; ^#^represents outcomes significantly different from negative control; ^+^represents outcomes significantly different from chloroquine-treated group; ^&^represents significantly different from Coartem-treated group. ARF represents the aqueous residual fraction; EAF represents the ethyl acetate fraction; EF represents the ethanolic fraction

The data presented in Fig. 7A show a significant increase (*p* < 0.0001) in the white cell count of animals infected with *P. bergehi* (4.55 ± 0.21) in comparison with the control (14.50 ± 2.1). This alteration was significantly ameliorated by treatment with chloroquine (3.9 ± 0.14, *p* < 0.0001), Coartem (9.35 ± 0.25, *p* = 0.013), EAF 400 mg/kg (9.05 ± 0.79, *p* = 0.009) and EF 400 mg/kg (10.0 ± 0.40, *p* = 0.03). The percentage of lymphocytes was also significantly increased (*p* = 0.03) in animals infected with *P. berghei (*76.0 ± 4.00*)* compared to the control animals (56.50 ± 2.12). Treatment with chloroquine (60.00 ± 0.0, *p* = 0.07), Coartem (63.00 ± 9.8, *p* = 0.19), ARF 100 mg/kg (69.00 ± 1.4, *p* = 0.78), EAF 400 mg/kg (62.50 ± 3.53, *p* = 0.16) and EF 400 mg/kg (65.00 ± 0.0, *p* = 0.33) caused a slight decrease in the percentage of lymphocytes in the infected animals (Fig. 7B). As shown in Fig. 7C, in contrast to the observed percentage of lymphocytes, malarial infection resulted in a significant decrease in neutrophils (21.50 ± 4.95, *p* = 0.03) compared with the control (42.00 ± 2.28), which was significantly improved by treatment with chloroquine (44.45 ± 6.36, *p* = 0.01).

#### Effect of *C. odorata* fractions on platelet indices of malaria-infected mice

**Fig. 8:**
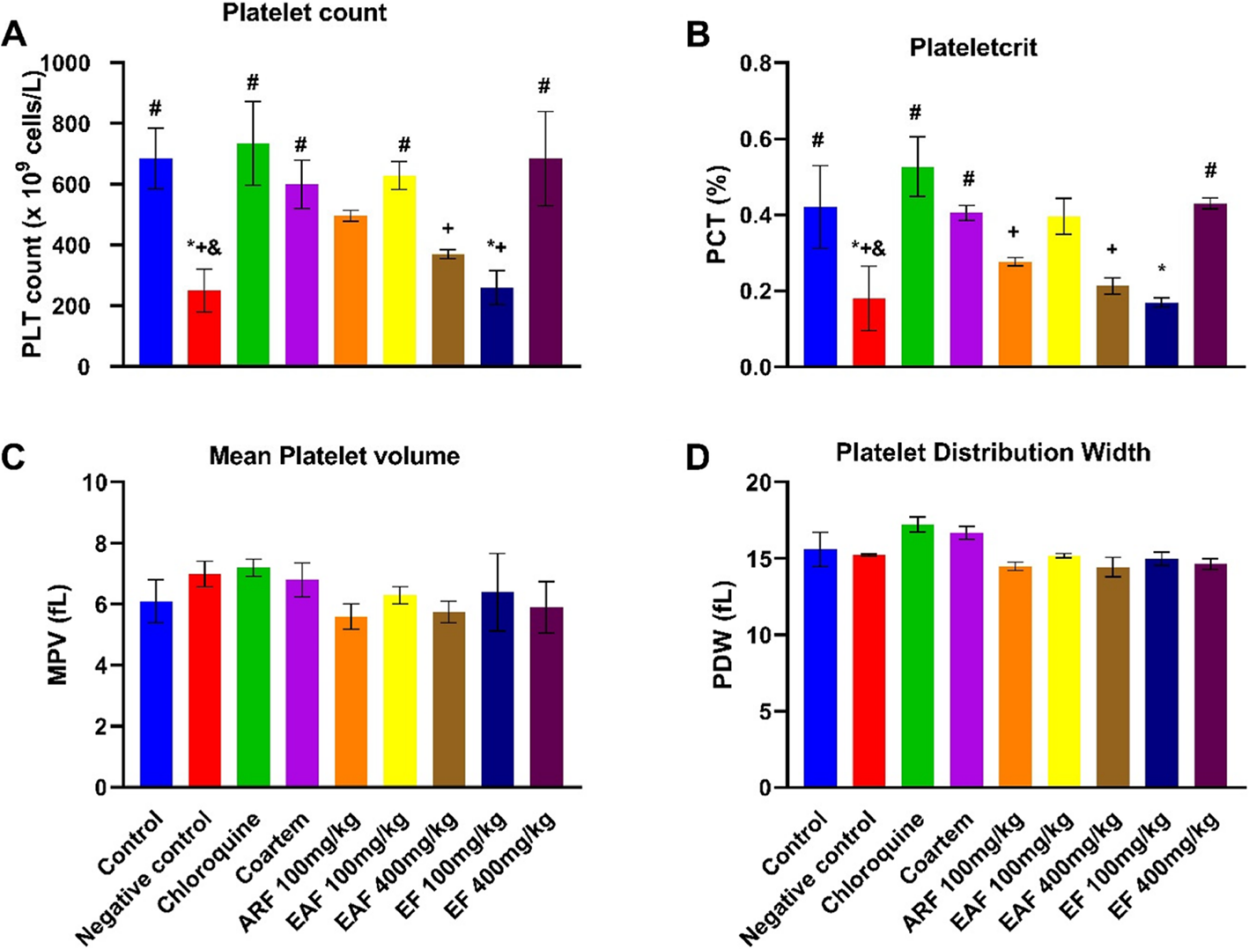
Effect of standard drugs and *C. odorata* fractions on the platelet indices of *P. berghei*-infected mice. The data are the mean of three replicates ± standard deviation (SD). All measures were considered significant at *p* < 0.05. ^*^ represents outcomes significantly different from control; ^#^represents outcomes significantly different from negative control; ^+^represents outcomes significantly different from chloroquine-treated group; ^&^represents significantly different from Coartem-treated group. ARF represents the aqueous residual fraction; EAF represents the ethyl acetate fraction; EF represents the ethanolic fraction

The data presented in Fig. 8A show that the platelet counts of animals infected with malaria parasites were significantly reduced relative to the control. However, the effects were significantly improved by treatment with chloroquine, Coartem, 100 mg/kg EAF and 400 mg/kg EF. Similarly, the plateletcrit of animals infected with malaria parasites was significantly reduced compared with the control (Fig. 8B). Treatment with chloroquine, coartem and EAF 400 mg/kg significantly ameliorated the PCT reduction. There was no significant alteration in the mean platelet volume (Fig. 8C) or the platelet distribution width (Fig. 8D) following infection with the malaria parasite.

### Chemical fingerprint of the ethanolic fraction of *C. odorata* aqueous leaf extract

**Fig. 9:**
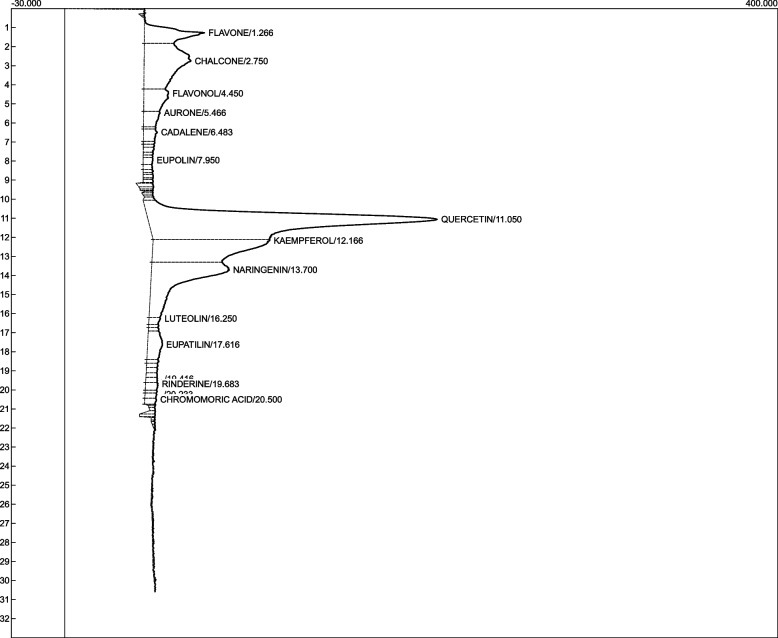
Chromatogram of the HPLC screening of the ethanolic fraction of *C. odorata* aqueous leaf extract

HPLC screening of the ethanolic fraction of the *C. odorata* aqueous extract identified thirteen bioactive components, with quercetin (41.5%), kaempferol (15.9%), naringin (14.4%) and chalcone having the highest concentrations (Fig. 9 and Table 3).

**Table 3:** Chemical composition of the ethanol fraction of the aqueous extract of* C. odorata*

S/n	Composition	Retention Time	Area%	Molecular weight g/mol	Chemical structure
1	Flavone	1.26	5.1	222.239	
2	Chalcone	2.75	11.7	208.2552	
3	Flavonol	4.45	3.6		
4	Aurone	5.47	1.6	222.239	
5	Cadalene	6.48	1.1	198.303	
6	Eupolin	7.95	0.5		
7	Quercetin	11.05	41.5	302.23	
8	Kaempferol	12.17	15.9	286.236	
9	Naringenin	13.70	14.4	272.2528	
10	Luteolin	16.25	0.6	286.236	
11	Eupatilin	17.62	2.7	344.315	
12	Rinderine	19.68	0.7	299.363	
13	Chromomoric acid	20.50	0.5	308.4	

## Discussion

Malaria is a global health challenge with endemicity in sub-Saharan Africa, where there are multiple drug-resistant strains and limited access to modern health care facilities, especially in rural areas [1, 2]. These factors underscore efforts to integrate botanical antimalarial molecules into the health care system, as this could facilitate sustainable development goal 3.8, which targets attaining universal health coverage for all [6]. Considering this, the present study evaluated the curative antimalarial properties of *C. odorata*, a relatively abundant but underexplored plant in Africa.

The quantitative phytochemical screening of the aqueous crude extract of *C. odorata* and its solvent fractions revealed richness in flavonoids rather than phenols, as observed from the abundance of quercetin, kaempferol, naringenin and luteolin in the ethanolic fraction of the plant extract. This finding corresponds with previous studies, which suggest that the methanolic leaf extract of *C. odorata* contains higher concentrations of flavonoids than phenolics [35]. The lethality study conducted showed that the median lethal dose of the crude extract was higher than 2000 mg/kg. However, due to the extremely low yield of the aqueous residual fraction obtained during solvent partitioning (8.66%), the highest dosage for this fraction was pegged at 100 mg/kg to prevent any adverse reactions in the animals.

Furthermore, our findings in this study revealed that the ethyl acetate, ethanolic and aqueous residual fractions of the aqueous extract of *C. odorata* possess highly active antimalarial properties with a percentage of chemo-suppression between 75.8–97.6%. According to Gathirwa et al. [36] and Afolayan et al. [37], botanical samples showing chemo-suppression of the *Plasmodium* parasite of 60% or more are strongly active, 30% to 60% are moderately active, and 30% or less are weakly active. Among the plant extracts, treatment with 400 mg/kg ethanolic fraction resulted in the highest decrease in parasite load (97.6%), which was comparable to the activities of the conventional drugs chloroquine (98.6%) and Coartem (98.8%). A similar study conducted by Nworgu et al. [22] revealed that 250, 500 and 1000 mg/kg ethanolic extracts of *C. odorata* induced 84.81%, 90.70%, and 95.63% reductions in the parasite burden of mice infected with *P. berghei,* respectively.

The anti-malarial activities elicited by the solvent fractions of *C. odorata* leaf extract is likely due to the abundance of flavonoids such as quercetin, kaempferol, naringenin and luteolin. Quercetin and kaempferol has been reported to possess moderate anti-malarial activity which is elicited by the compounds ability to downregulate the activity of tumour necrotic factor -α, thus, reducing malaria severity and malaria-associated tissue damage [38–40].

Malaria infection and subsequent treatment have been associated with significant alterations in haematological parameters [11–13, 41]. The results of this study showed that mice infected with the chloroquine-resistant strain of *P. berghei* had considerably lower levels of anaemia-related indices: haemoglobin count, haematocrit level, and red blood cell count. According to the study conducted by Mwaiswelo et al. [41], most adult and paediatric patients infected with *P. vivax* and *P. falciparum* frequently have low RBC, haematocrit, and haematology counts. Similar haematological alterations in have also been observed in mouse models [11–13]. The Plasmodium spp. elicits its infectious activity by invading red blood cells causing the premature destruction of RBCs resulting in anaemia [42]. In this study, treatment with the different solvent fractions of *C. odorata* mitigated the alterations in the red blood cell indices of the infected animals which suggests that the solvent fractions of *C. odorata* elicited its anti-malarial activity by preventing the invasion of red blood cells by malarial parasite.

Malaria infection also significantly increased the white blood cell count and percentage of lymphocytes and decreased the percentage of neutrophils. This is similar to the study conducted by Ounjaijean et al. [43], which showed that WBC counts are elevated in mice infected with *P. berghei.* The increased lymphocytes seen in the current study could be a result of the immune system's efforts to combat the malaria parasite. On the other hand, a depletion of neutrophils may indicate compromise of the immune system by malaria infection, as neutrophils are involved in the phagocytosis of *Plasmodium spp.* [44]. The mitigative effect of treatment of malarial infected mice with the solvent fractions of *C. odorata* suggests that the plant inhibits the proliferation of *plasmodium* spp. in the infected host which reduces the immune responses associated with malaria infection.

Platelets have been identified as one of the major host defence systems against the malaria parasite [45]. The current study noted a substantial decline in platelet and plateletcrit counts in *P. berghei*-infected animals. Series of literature have shown that platelet counts is usually depleted in both humans and mouse models during infection with *Plasmodium spp.* [46–48]. According to the studies conducted by Zhou et al. [49], infection with *Plasmodium spp.* in mice inhibits the secretion of anti-platelet antibody which prevents the destruction of the malaria parasite by the platelet sells of the host organism which explains the depletion in the platelet count of the mice infected with *P. berghei* as observed from this study. The improvement of these haematological alterations following administration of the various fractions of the aqueous extract of *C. odorata,* particularly the ethanolic fraction at 400 mg.kg, suggests that the bioactive compounds of the plant extract and fraction could have the ability to stimulate platelet production or prevent the production of anti-platelet antibody during malaria infection.

This could be due to the synergistic interactions among the bioactive components of the extract. HPLC screening of the ethanolic fraction revealed that the extract was most abundant in quercetin, kaempferol, naringenin and chalcone. Numerous studies have associated the antimalarial activities of botanical drugs with the presence of quercetin and kaempferol [38, 50–52].

## Conclusion

The findings from the current study suggest that *C. odorata* fractions could be used as an adjuvant and/or alternative to treat malaria, particularly in sub-Saharan Africa, where most of the population cannot afford or do not have access to primary health care services. The ethanolic fraction of *C. odorata* had a comparative antimalarial activity to standard drugs chloroquine and artermether-lumenfantrine, which suggests that the fraction contains bioactive molecules with potent anti-malarial properties. The fractions of *C. odorata* leaf extract also mitigated the haematological alterations induced by malaria infection. Additionally, due to synergistic interaction between among the bioactive compounds in the fractions using botanical drug may be a more effective treatment option given that the malaria parasite is constantly evolving and acquiring resistance to available drugs. The synergistic effects of these bioactive components could facilitate attack of multiple parasitic pathways, making it difficult for the parasite to survive within its host.

## Data Availability

All data generated or analysed during this study are included in this published article.
